# Failure of Oral Risedronate Therapy to Prevent Spontaneous Vertebral Fracture in a Patient Ceasing Denosumab: A Cautionary Case

**DOI:** 10.1002/jbm4.10396

**Published:** 2020-08-21

**Authors:** Dahlia F Davidoff, Christian M Girgis

**Affiliations:** ^1^ Department of Diabetes and Endocrinology Royal North Shore Hospital Sydney New South Wales Australia; ^2^ Department of Diabetes and Endocrinology Royal North Shore Hospital, University of Sydney Sydney New South Wales Australia; ^3^ Department of Diabetes and Endocrinology Westmead Hospital Sydney New South Wales Australia

**Keywords:** ANTIRESORPTIVES, BISPHOSPHONATES, DENOSUMAB, OSTEOPOROSIS, RISEDRONATE, SPONTANEOUS VERTEBRAL FRACTURE

## Abstract

Denosumab is a highly effective treatment for postmenopausal osteoporosis, significantly improving BMD and reducing risk of fracture. However, denosumab's effect is transient with the risk of a rebound increase in bone turnover following withdrawal of this potent RANKL inhibitor. This poses challenges, particularly in individuals seeking to discontinue denosumab, such as those experiencing a direct complication of prolonged antiresorptive therapy or those in whom an antiresorptive drug holiday would be ordinarily considered. Bisphosphonate strategies to mitigate postdenosumab bone loss are being actively studied. We describe the case of a 73‐year‐old woman who developed a spontaneous vertebral fracture following denosumab discontinuation, despite prolonged treatment with bisphosphonate therapy both before her course of denosumab (20 years of use) and following denosumab discontinuation (1 year of use). This is a cautionary case seeking to highlight uncertainties around the safe withdrawal of denosumab therapy despite intervening treatment with bisphosphonates. © 2020 The Authors. *JBMR Plus* published by Wiley Periodicals, Inc. on behalf of American Society for Bone and Mineral Research © 2020 The Authors. *JBMR Plus* published by Wiley Periodicals LLC on behalf of American Society for Bone and Mineral Research.

## Introduction

Denosumab is a human IgG2 monoclonal antibody to RANKL.^(^ ^1^ ^)^ By binding to RANKL, denosumab prevents the RANKL/RANK receptor interaction on osteoclasts, resulting in reduced osteoclast formation and function.^(^ ^1^ ^)^ Denosumab has revolutionized the management of postmenopausal osteoporosis because of its dramatic impact on BMD and its reduction of fracture risk.^(^ ^2^ ^)^ Current guidelines recommend the consideration of an intermission in antiresorptive therapy after prolonged treatment following careful evaluation of patient characteristics, BMD, and prior history of fracture.^(^ ^3^ ^)^ The risk of atypical femur fracture (AFF) in patients on antiresorptive therapy is time‐dependent, with the risk increasing steadily after 5 years of treatment.^(^ ^4^ ^)^ Patients on prolonged denosumab therapy,^(^ ^5^ ^)^ particularly those on lengthy prior treatment with bisphosphonate,^(6^ ^)^ are also at risk of AFF. We describe a case of a postmenopausal woman who had received a quarter century of antiresorptive therapy, first with oral bisphosphonate therapy for 20 years, followed by 5 years of denosumab. After careful consideration, she was transitioned from denosumab to an oral bisphosphonate in an effort to initiate an antiresorptive treatment intermission. Despite cautious treatment with a bisphosphonate and her many years of preceding bisphosphonate treatment, she sustained a spontaneous single vertebral fracture 13 months after denosumab discontinuation.

## Clinical Vignette

A 73‐year‐old woman with a lengthy history of osteoporosis and an extensive fracture history received 25 years of treatment with antiresorptive therapy. For the first 20 years, she received alendronate, followed by denosumab for 5 years. Because of her prolonged history of antiresorptive use, in the absence of recent minimal trauma fractures and with improved BMD values performed by DXA (lumbar spine density, 0.885 g/cm^2^, *T*‐score, 1.5 SD; left femoral neck density, 0.613 g/cm^2^, *T*‐score, 2.1 SD; left total hip, 0.683 g/cm^2^, *T*‐score, 2.1 SD), an antiresorptive treatment holiday was planned. She was subsequently transitioned from denosumab to a weekly oral bisphosphonate risedronate to protect from a rebound loss of bone density.^(^ ^7^, ^8^ ^)^ Of note, she had a remote history of vertebral and pelvic fractures occurring over three decades, but not recently. A bone scan prior to transition to risedronate showed non‐avid old vertebral fractures. Her lengthy prior treatment with bisphosphonate was also considered to potentially alleviate a rebound increase in bone turnover postdenosumab withdrawal. Six months after switching from denosumab to the oral bisphosphonate, bone turnover markers reassuringly remained within the reference range with P1NP 35 μg/L (15 to 115) and CTx 360 ng/L (100 to 1000) as assessed with a Roche cobas immunoassay analyzer (Roche Diagnostics, Mannheim, Germany).

**Fig 1:**
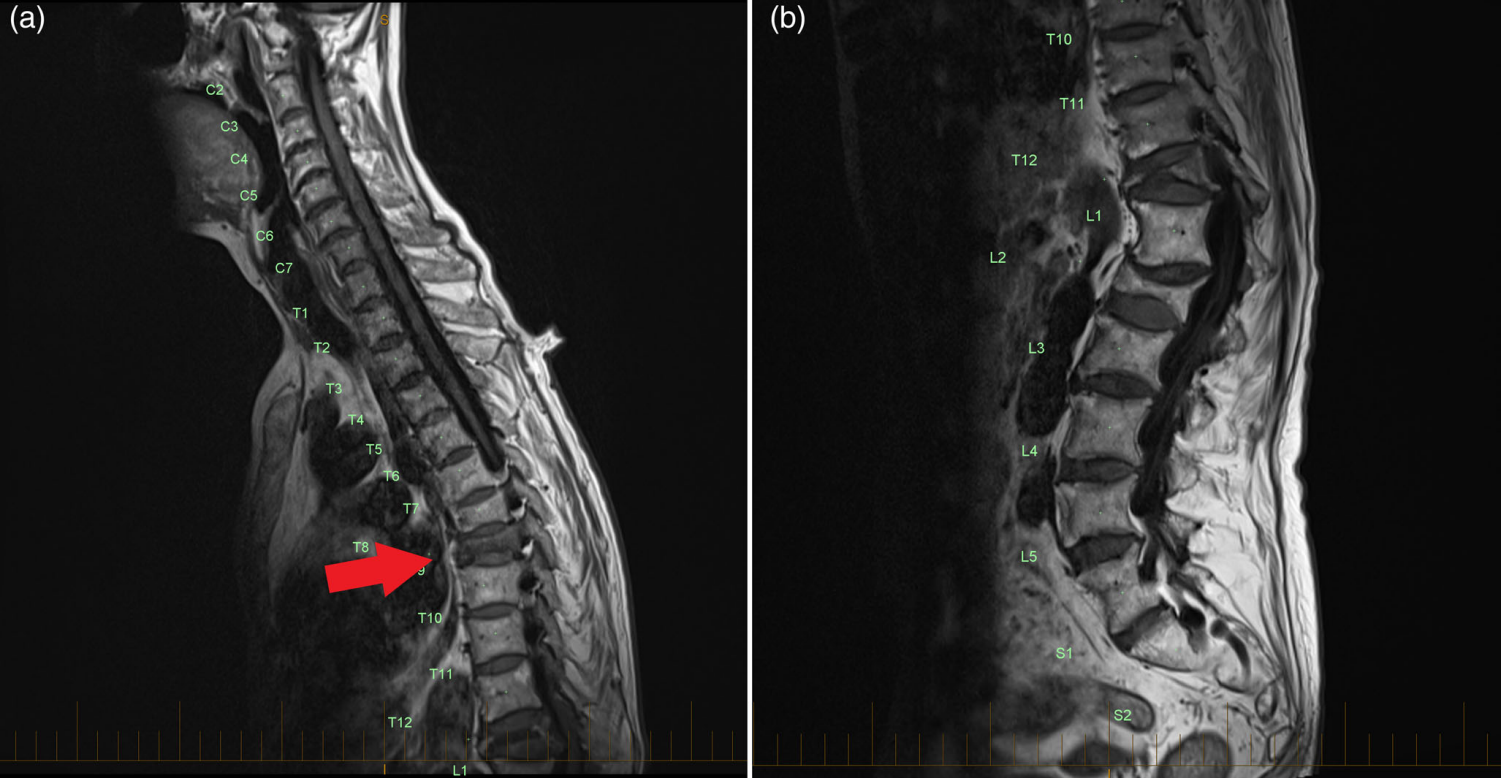
T_1_‐weighted MRI imaging of the thoracic (*A*) and lumbar spine (*B*) showing an acute compression fracture identified in T8 (arrow) and evidence of prior vertebral compression fractures in T7, T12, and L2.

**Fig 2:**
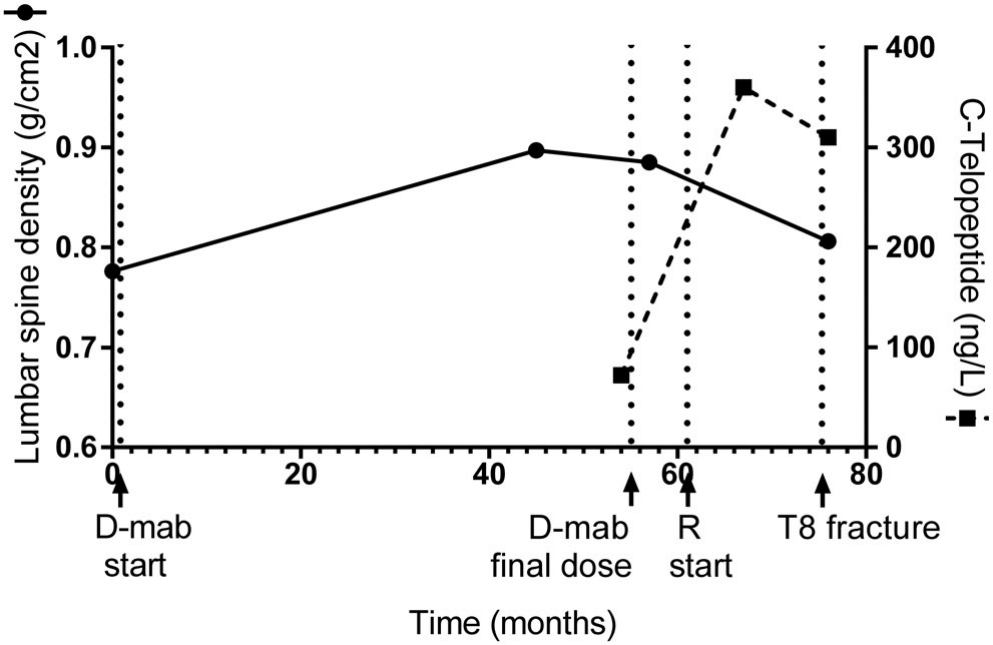
BMD and bone turnover marker response to denosumab and risedronate. D‐mab = denosumab; R = risedronate.

Thirteen months after transitioning to the oral bisphosphonate, the patient sustained a T8 minimal trauma fracture after lifting a heavy metal pan (Fig. 1A and 1B). The patient reported compliance to risedronate. Bone turnover marker analysis performed 6 weeks after the fracture were stable compared to previous results with P1NP 38 μg/L (15 to 115) and CTx 310 ng/L (100 to 1000). A secondary osteoporosis screen was unremarkable including replete 25‐hydroxyvitamin D of 134 nmol/L (50 to 140) and normal corrected calcium of 2.44 mmol/L (2.15 to 2.55), serum phosphate of 1.31 mmol/L (0.8 to 1.5), intact PTH of 20 ng/L (15 to 68), thyroid‐stimulating hormone of 0.70 mIU/L (0.40 to 5.00), negative celiac serology by deamidated gliadin and tissue transglutaminase IgA and IgG, and normal serum protein electrophoresis and immunofixation. However, a DXA showed a decline in lumbar spine bone density (0.806 g/cm2, *T*‐score, 2.2; 8.9% decline over 19 months) and minor reductions in the left total hip left femoral neck density (Fig. 2, Table 1). Given the decline in BMD, denosumab treatment was resumed and will continue indefinitely.

**Table 1:** Change in Lumbar Spine BMD, CTx, and P1NP Over Time

Months from baseline	Lumbar spine density (g/cm^2^)	Lumbar spine *T*‐score	Lumbar spine density (% change) from baseline	CTx (ng/L)	Total P1NP (μg/L)
0	0.776	−2.5	NA	‐	‐
45	0.897	−1.4	15.6%	‐	‐
54	‐	‐	‐	72	20
57	0.885	−1.5	14.0%	‐	‐
67	‐	‐	‐	360	35
76	0.806	−2.2	3.9%	310	38

## Discussion

Denosumab is a highly effective treatment for postmenopausal osteoporosis, which results in continued increases in BMD over time^(^ ^9^ ^)^ and is well‐tolerated.^(^ ^10^ ^)^ In the Freedom Extension [Clinicaltrails.gov: NCT00089791 (FREEDOM) and NCT00523341 (Extension): An Open Label, Single Arm, Extension Study to Evaluate the Long Term Safety and Sustained Efficacy of Denosumab (AMG162) in the Treatment of Postmenopausal Osteoporosis] Trial, the mean change in lumbar spine BMD from baseline to 5 years was +13.7%, comparable to our patient's improvement and by 10 years, up to +21.7%.^(^ ^9^ ^)^ However, denosumab withdrawal is problematic with a risk of rapid loss in bone density gained^(^ ^11^, ^12^, ^13^ ^)^ and a risk of vertebral fractures, especially in those with prior vertebral fracture.^(^ ^13^, ^14^, ^15^ ^)^

Strategies for mitigating postdenosumab bone loss are under examination, but the literature suggests that an oral bisphosphonate may be the most indefinitely approach at this time.^(^ ^7^, ^8^ ^)^ Post‐FRAME (Fracture Study in Postmenopausal Women with Osteoporosis) Trial data found that following denosumab cessation, treatment with oral risedronate (*n* = 5) provided a 41% retention of the increase in BMD achieved with denosumab.^(^ ^7^ ^)^ The DAPS (Denosumab Adherence Preference Satisfaction ) Study further addressed the question of transition to an oral bisphosphonate with a 24‐month open‐label randomized cross‐over study.^(^ ^8^ ^)^ A majority of postmenopausal women with osteopenia who switched to alendronate after 24 months of denosumab maintained or gained bone density in the DAPS Study in the lumbar spine, total hip, and femoral neck (84.1%, 92.4%, and 78.3% of participants, respectively).^(^ ^8^ ^)^ Bisphosphonates may vary in the potency of their antiresorptive effect and the CTx rise following denosumab may be mitigated by a potent bisphosphonate, whereby alendronate may be more effective than risedronate.^(^ ^16^ ^)^ Studies of BMD retention in patients who switched from denosumab to zoledronic acid have been conflicting. A series of six postmenopausal women found a lack of bone density retention in patients transitioned to zoledronic acid,^(^ ^17^ ^)^ whereas post‐FRAME data showed that the 11 women who switched to zoledronic acid had 73% retention of the gains in BMD after 1 year.^(^ ^7^ ^)^

The rapid loss of bone postdenosumab is not uniform among denosumab users, and the risk is considered greatest in patients who were bisphosphonate‐naïve prior to denosumab,^(^ ^18^, ^19^ ^)^ and those who achieved a marked increase in BMD on denosumab treatment.^(^ ^8^ ^)^ Optimal bisphosphonate treatment strategies to mitigate postdenosumab bone loss are under investigation.^(^ ^20^ ^)^

In our patient, two decades of prior treatment with a bisphosphonate and a postdenosumab course of risedronate failed to prevent a rebound vertebral fracture following denosumab cessation. The vertebral fracture was speculated to relate to a rebound phenomenon postdenosumab in light of the bone density decline and increase in bone turnover markers following denosumab cessation. Interestingly, our patient's CTx following denosumab was not particularly high, and fell within the reference range for postmenopausal women. Our management was consistent with current recommendations to consider an intermission in antiresorptive therapy after prolonged treatment, in individuals who do not exhibit a high fracture risk, those without recent fracture, or with *T*‐score > − 2.5 SD, and to consider bisphosphonate transition postdenosumab withdrawal.^(^ ^3^ ^)^ The aim of an antiresorptive “drug holiday” is to reduce the risk of medication adverse events such as atypical femoral fracture.^(^ ^3^ ^)^ Nonetheless, the patient still fractured postdenosumab.

This case highlights the uncertain efficacy of bisphosphonate use to adequately suppress bone turnover following denosumab discontinuation and raises three important points: (i) Patients with previous history of vertebral fracture, no matter how remote, are at greatest risk of postdenosumab spontaneous vertebral fractures despite bisphosphonate use; (ii) standard markers of bone turnover may not adequately herald the onset of spontaneous vertebral fractures or decline in bone density following denosumab; and (iii) the dynamics of bone loss following denosumab withdrawal are poorly understood and unpredictable. Until further research emerges regarding the safe withdrawal of denosumab in patients on long‐term antiresorptive treatment, a high degree of caution is necessary. An individualized assessment of the risk: benefit ratio of long‐term treatment with denosumab is required, bearing in mind that this an otherwise highly effective fracture‐preventing treatment.

## Disclosures

There are no disclosures.

### Peer Review

The peer review history for this article is available at https://publons.com/publon/10.1002/jbm4.10396.
